# Frequent pulse disturbances shape resistance and resilience in tropical marine microbial communities

**DOI:** 10.1038/s43705-023-00260-6

**Published:** 2023-06-06

**Authors:** Winona Wijaya, Zahirah Suhaimi, Cherlyn Xin’Er Chua, Rohan Shawn Sunil, Sandra Kolundžija, Ahmad Muzakkir Bin Rohaizat, Norzarifah Binti Md. Azmi, Nur Hazlin Hazrin-Chong, Federico M. Lauro

**Affiliations:** 1 Asian School of the Environment, Nanyang Technological University, Singapore, Singapore; 2 Department of Anthropology, University of California Santa Cruz, Santa Cruz, CA USA; 3 Center for Southeast Asian Coastal Interactions, Santa Cruz, CA USA; 4 School of Biological Sciences, Nanyang Technological University, Singapore, Singapore; 5 Department of Biosciences, Universiti Teknologi Malaysia, Skudai, Johor Bahru Malaysia; 6 Department of Biological Sciences and Biotechnology, Universiti Kebangsaan Malaysia, Bangi, Selangor Malaysia; 7 Singapore Centre for Environmental Life Sciences Engineering (SCELSE), Nanyang Technological University, Singapore, Singapore

**Keywords:** Microbial ecology, Population dynamics

## Abstract

The Johor Strait separates the island of Singapore from Peninsular Malaysia. A 1-kilometer causeway built in the early 1920s in the middle of the strait effectively blocks water flowing to/from either side, resulting in low water turnover rates and build-up of nutrients in the inner Strait. We have previously shown that short-term rather than seasonal environmental changes influence microbial community composition in the Johor Strait. Here, we present a temporally-intensive study that uncovers the factors keeping the microbial populations in check. We sampled the surface water at four sites in the inner Eastern Johor Strait every other day for two months, while measuring various water quality parameters, and analysed 16S amplicon sequences and flow-cytometric counts. We discovered that microbial community succession revolves around a common stable state resulting from frequent pulse disturbances. Among these, sporadic riverine freshwater input and regular tidal currents influence bottom-up controls including the availability of the limiting nutrient nitrogen and its biological release in readily available forms. From the top-down, marine viruses and predatory bacteria limit the proliferation of microbes in the water. Harmful algal blooms, which have been observed historically in these waters, may occur only when there are simultaneous gaps in the top-down and bottom-up controls. This study gains insight into complex interactions between multiple factors contributing to a low-resistance but high-resilience microbial community and speculate about rare events that could lead to the occurrence of an algal bloom.

## Introduction

Microbial ecologists have been fascinated by the impact of disturbances on microbial community succession, resistance, and resilience for several decades. Although multiple definitions of these terms have been proposed, it is generally agreed that resistance refers to communities’ capability to remain stable despite disturbances, and resilience refers to their ability to return to that state after a disturbance-induced shift [1–3]. Many studies, both observational and experimental, have been conducted on disturbance and resilience, with several recent and comprehensive reviews that provide a good overview on the topic [1–5]. This wealth of research has yielded outcomes that might seem conflicting at first glance. However, the apparent discrepancies just emphasise the intricate nature of the natural communities, which exhibits varying responses depending on the specific characteristics and pace of each disturbance event.

The city-island-state of Singapore is bordered by two Straits: the Singapore Strait on the southern side separates Singapore from the Riau Islands of Indonesia, while the Johor Strait on the northern side separates Singapore from Peninsular Malaysia. In the 1920s, a 1-kilometer causeway was built on the Johor Strait to connect British Malaya with Singapore, accelerating the area’s economic development. The causeway was built in the middle of the Strait, with years of debris and disrepair preventing water from flowing to either side. As a result, the waters in the inner part of the strait have low turnover rates and nutrient build-up from various anthropogenic inputs [6–10]. The Johor Strait is often experiencing eutrophic conditions, with concentration of nutrients (especially nitrogen) often higher than 10 µM for NO_X_ and 40 µM for NH_4_, 1 order of magnitude higher than that of the Singapore Strait [11].

Ecologically, a community is governed both from higher trophic levels (presence of predators or consumers) and lower trophic levels (availability of nutrients or food) [12–15]. This is more commonly known as the top-down and bottom-up control, respectively. No natural microbial community is immune to changes in environmental conditions, and the microbial communities of the inner Johor Strait are no exception. The Johor Strait is home to many open cage fish farms, and seasonal harmful algal blooms (HABs) have occurred in these waters, causing billions of dollars in losses to the aquaculture industry [16–21]. However, despite the nutrient load at levels favourable to the inception of HABs, the microbial community in Johor Strait is under some sort of dynamic stability as the water does not experience blooms every day. While many observational studies on both bloom and baseline conditions have been conducted in recent years, none of these studies were done from the perspective of disturbance/resilience of a microbial community despite mentioning some of these disturbances (e.g., sporadic and sudden changes in nutrient inputs and salinity) [6, 8, 9, 17–21]. Moreover, as the Johor Strait is influenced more by short-term environmental changes rather than seasonal ones [11], the aforementioned studies were not conducted in a high enough temporal resolution to see the influences of the multitude of pulse disturbances on the microbial community of the Johor Strait.

In this study, we analyse the short-term drivers that affect the Johor Strait microbial populations. We focused on the community compositional resilience (i.e., how the microbial composition remains stable despite disturbances), in contrast to functional resilience (i.e., how the same functions are retained even when the microbial composition changes). We sampled water every other day from four sites in the Johor Strait, sequenced the 16 S rRNA gene amplicons, concurrently analysing various water quality parameters and tidal conditions. We posit that, in response to repeated pulse disturbances, the microbial community evolves to respond to new disturbances with low resistance and high resilience, which influence how and when blooms might develop in the Johor Strait.

## Methods

### Data collection

Water sampling and data collection was conducted at the inner Eastern side of the Johor Strait. Sampling sites were chosen due to their accessibility to the Johor Strait waters, either on jetties or land parcels that protrude into the Strait, as shown in Fig. S1 and Table S1. Sample and data collection was conducted from 4 November through 28 December 2020, consistently on Monday, Wednesday, Friday, and Saturday inclusive of these dates (except for samples SNB23 and STL23 on 14 December 2020 due to equipment breakdown).

At each site, surface water samples were collected at 1 m depth using a portable pump and filtration system (OSMO) [22]. For DNA extraction, 1.5 L of 150 µm pre-filtered water were filtered through sterile 0.22 µm polyether-sulfone Sterivex filter units (Merck Millipore, Darmstadt, Germany), after which approximately 2 mL RNA*later* (Sigma Aldrich, Darmstadt, Germany) was added into the Sterivex, then stored in a dry shipper charged with liquid nitrogen. Duplicate Sterivex samples were collected for DNA extraction. Sample processing took at most 20 minutes between the start of water collection until the addition of RNA*later*.

Temperature and salinity of the water was measured onsite (Extech Instruments, Nashua, New Hampshire, USA). For dissolved inorganic nutrient analysis, 12 mL of water from the Sterivex filtrate was collected into acid-washed polypropylene centrifuge tubes, then flash-frozen in liquid nitrogen. Flow cytometry samples were collected from the 150 µm pre-filtrate, fixed with electron microscopy grade glutaraldehyde (0.5% v/v final concentration, Sigma Aldrich) at 4 °C in the dark before flash-frozen in liquid nitrogen [23, 24]. Chlorophyll samples for two size fractions were obtained: completely unfiltered water, and *<*150 µm from the pre-filtrate. For each size fraction, 50 mL of water sample was filtered slowly in a dropwise fashion on a 25 mm glass fiber filter (Whatman GE Healthcare Life Sciences, Buckinghamshire, UK), then individually wrapped with aluminium foil and flash-frozen in liquid nitrogen.

After each sampling trip, Sterivex filters, cryovials, and chlorophyll glass fiber filters were stored at −80 °C whilst water samples for nutrient analysis were stored at −20 °C until sample processing and analysis.

#### Tide and rain data acquisition

Half-hourly tide measurements were acquired from the Maritime and Port Authority of Singapore (https://www.mpa.gov.sg/) from the government-run Sembawang Tide station (1.465° N, 103.835° E) (Table S2). Current strength and direction were calculated by the slope of the tidal data, i.e., the change in tide height over a unit of time. Dates of peak neap and spring tides were estimated from moon phases, which were then used to calculate the number of days between sampling and its nearest peak of neap/spring tide (Table S3). Rain data was acquired from the Meteorological Service Singapore (http://www.weather.gov.sg/climate-historical-daily/) using Sembawang Station (1.4252° N, 103.8202° E) for eastern sampling sites and Admiralty Station (1.4439° N, 103.7854° E) for western sampling sites.

### Sample processing

#### Dissolved inorganic nutrient concentration

Samples for dissolved inorganic macronutrients were thawed at room temperature and immediately measured on a SEAL AA3 High-Resolution AutoAnalyser (SEAL Analytical, Norderstedt, Germany). The nutrients measured were phosphate (PO_4_), silicate (Si), ammonia (NH_4_), nitrite (NO_2_), and calculated nitrate (NO_3_), all in *µ*mol/L. Total Dissolved Inorganic Nitrogen (DIN) was calculated as NO_2_ + NO_3_ + NH_4_.

#### Chlorophyll concentration

GF/F filter of each sample was placed into acid-washed centrifuge tubes containing 90% molecular-grade acetone and incubated in the dark at 4 °C overnight. Samples were then centrifuged at 500 × *g* for 10 min. The supernatant (2 mL) was then aliquoted into a disposable cuvette for measurement using the FluoroMax spectrophotometer (Horiba).

#### Flow cytometry (FCM)

Our FCM analysis protocol was optimised for counting viruses based on Brussaard (ref. 23) and Brussaard et al. (ref. 24). Briefly, glutaraldehyde-fixed samples were diluted using Tris-EDTA buffer and stained using SYBR Green I (Invitrogen, Life Technologies, Eugene, Oregon, USA). Samples were run at a slow flow rate (approximately 10 µL/s) on the CytoFLEX benchtop flow cytometer (Beckman Coulter, Brea, California, USA) equipped with blue (488 nm) and violet (405 nm) lasers specifically to distinguish virus particles. Virus and bacterial populations were gated using the CytExpert software (Beckman Coulter), validated against fluorescent microscopy counts and serially diluted positive controls (data not shown). The raw FCM data was then processed further in the R statistical environment [25] using the package *phenoflow* [26].

#### DNA extraction, amplification, sequencing, and data processing

DNA samples were extracted in random order to minimise bias and batch effects. We followed the default factory protocols of the DNEasy Powersoil Pro kit (QIAGEN, Germantown, Maryland, USA) with a few modifications. Firstly, filter units were rinsed with 1X phosphate-buffered saline to remove the RNA*later*. The Sterivex casing was broken to expose the filter inside, which was inserted into Powersoil Pro Bead tubes (prepared as per the first step of the default protocols), then incubated at 70 °C, 500 rpm for 15 min, twice. Molecular grade Phenol-Chloroform-Isoamyl Alcohol (25:24:1 v/v) (Sigma Aldrich) was added into the bead tubes, after which the samples were subjected to rest of the factory protocol starting at the 10-minute vortexing step. DNA was eluted into 60 *µ*L of nuclease-free water and quantified using a Qubit 2.0 fluorometer (Invitrogen, Life Technologies).

For each sample, PCR was done in triplicates using KAPA HiFi HotStart ReadyMix (Roche, Cape Town, South Africa) with primers that have the standard Nextera Illumina adapter attached at their 5ʹ end. The primer pair, 926WF (5ʹ-AAA-CTY-AAA-KGA-ATT-GRC-GG-3ʹ) and 1392 R (5ʹ-ACG-GGC-GGT-GTG-TRC-3ʹ) [27] was used specifically because it targets the V6-V8 hypervariable region of the SSU rRNA gene of all three bacteria, archaea, and eukarya at a considerably high coverage for environmental samples [28, 29]. After 22 cycles of amplification, triplicate amplicons of the same samples were pooled and then cleaned using KAPA HyperPure Beads (Roche).

Amplicon library preparation and sequencing was conducted at the sequencing facility of Macrogen APAC (South Korea). Briefly, a second round of PCR was done to attach dual barcodes to each sample for multiplexing. The pooled library was then sequenced on an Illumina MiSeq machine. The demultiplexed.fastq files were returned to us and further processed in the R statistical environment [25].

Adapter and primer sequences were removed from the reads using cutadapt (version 3.4) [30]. The reads were then subjected to processing using the DADA2 algorithm [31]. The SILVA rRNA database (version 132) [32] was used to classify the taxonomy of each amplicon sequencing variant (ASV), after which the PR2 database (version 4.14) [33] was used to re-classify the eukaryote ASVs. Afterwards, chlorophyll and arthropod sequences, as well as ASVs with less than 10 reads across samples were removed. Chlorophyll sequences were removed to avoid duplicate counts of photosynthetic organisms, whilst arthropods are usually bigger than 150 µm and thus the DNA detected most likely came from their body parts or eggs. ASVs below a total abundance of 10 reads might be an artifact of de-noising or wrongly read sequences. Afterwards, ASV abundances between technical duplicates were normalised and averaged. As shown in Fig. S2, the Bray-Curtis distance between different locations of the same day is generally larger than the distance between technical replicates.

### Statistical analysis

The R package *vegan* [34] was used in most statistical analyses of the processed sequencing data: calculation of alpha diversity indices (Shannon & Simpson indices), pairwise community dissimilarity using the Bray-Curtis distance, dimensionality reduction of these distances using non-metric multidimensional scaling (NMDS), analysis of environmental parameters using *envfit*, as well as Canonical Correspondence Analysis (CCA). For *envfit* analysis, all environmental parameters were used as input and only those with significant results (*p* < 0.005) were plotted. The packages *phyloseq* [35] and *ggplot2* [36] were also used to visualise the results of these analyses, and *ggpubr* [37] was used to perform statistical tests between different groups (Wilcoxon test for two groups, and Kruskal-Wallis test for multiple groups). Co-occurrence networks were built using the *SpiecEasi* algorithm [38] and analysed using *igraph* [39], all in the R environment. Networks were then visualised and exported using Gephi [40].

Principal Components Analysis (PCA) was conducted using the *princomp* function, while correlation analysis between environmental variables were conducted using the *cor* function. Visualisation of correlations were done using *corrplot* [41].

Distance-based phylogenetic trees were built to classify unclassified Saprospiraceae ASVs. In-silico PCR (https://github.com/egonozer/in_silico_pcr) was conducted on genomes downloaded from NCBI Refseq [42] with primers as per the above primer pair. Alignment was calculated using MAFFT [43] (Multiple Alignment using Fast Fourier Transform) using its ‘accurate’ algorithm (L-INS-i), and trees were constructed using FastTree [44], then visualised using FigTree (http://tree.bio.ed.ac.uk/software/figtree/). *E. coli* strain KS-12 was used as an outgroup to root the phylogenetic tree.

### Comparison with community dynamics over longer time periods

In a previous study, monthly sampling of the Johor Strait waters was conducted over the span of 2.5 years [11]. The Sembawang sampling station was found to be located less than 3.5 km away from the SBW station in this study. To validate the magnitude of the dynamic community resilience over longer periods of time, a total number of 25 SBW monthly samples previously acquired by Chènard et al. [11], were re-sequenced with the same library preparation and kit version, and then processed similarly to the methods described above. The R packages *vegan* [34], *phyloseq* [35], *ampvis2* [45], and *DESeq2* [46] were used in the statistical analysis and visualisation of the sequences. *Vegan* was used to perform NMDS, which was then visualised using *phyloseq* and *ggplot2*. Differential abundance of organisms between the monthly SBW samples (of Chènard et al. [11]) and bi-daily (every other day) SBW samples (of this study) before batch-effect correction was conducted using *DESeq2* and visualised with *ggplot2*. Batch-effect correction between the monthly and bi-daily SBW time-series was conducted using the *ComBat* function in the package *sva* [47] without taking into account covariates such as nutrient and rainfall data.

## Results

### General overview

During the course of sampling, the average salinity and temperature of the East Johor Strait waters were 24.2 and 30.2 °C, respectively. Approximately 35 rain days were recorded during the duration of sampling; the maximum number of days without rain was 4 days (29 Nov – 2 Dec 2020, inclusive). This trend is consistent with historical rain reports of Singapore (http://www.weather.gov.sg/climate-climate-of-singapore/).

Despite receiving approximately the same amount of rain as the western sites, the site closest to the mouth of the Strait (SBW) had a significantly higher average salinity at 25.54 (*p* = 0.0057, Fig. S3). The eastern sites were always affected by tidal mixing, while western sites were only mixed during spring tides. A higher community dissimilarity was observed closer to neap tides when tidal mixing was low (Fig. S4). Otherwise, there were no consistent patterns in the nutrient concentration and other observed environmental parameters between the sites. While the concentrations of dissolved nutrients did vary throughout the time series, variations were consistent across the four sites (Fig. S5).

### Amplicon Sequencing Results

From two technical replicates of the bi-daily data, a total of 27,543,728 sequencing reads were recovered overall. Meanwhile, resequencing of Chènard et al. [11] monthly samples gave us 3,700,114 raw reads. After trimming and denoising, a total of 5,575 ASVs were recovered in 119 bi-daily and 25 monthly samples, with an average of 43,029 reads per sample. Bacteria of the phyla Bacteroidetes Cyanobacteria, and Proteobacteria make up the majority of all organisms detected; while ASV0003 (*Roseobacter* HIMB11), ASV0004 and ASV0007 (cyanobacteria PCC6307) were the top three most abundant ASVs in the Johor Strait. Some past HAB taxa detected in previous studies conducted in the region [8, 16, 19–21, 48], were observed in our samples: dinoflagellates of genus *Chaetoceros* and family Kareniaceae, as well as diatoms of genus *Thalassiosira*. These taxa were only found in very low amounts at a mean of 83 reads per sample, however, they were present in all but 37 samples in our time series.

### Correlations

**Fig. 1:**
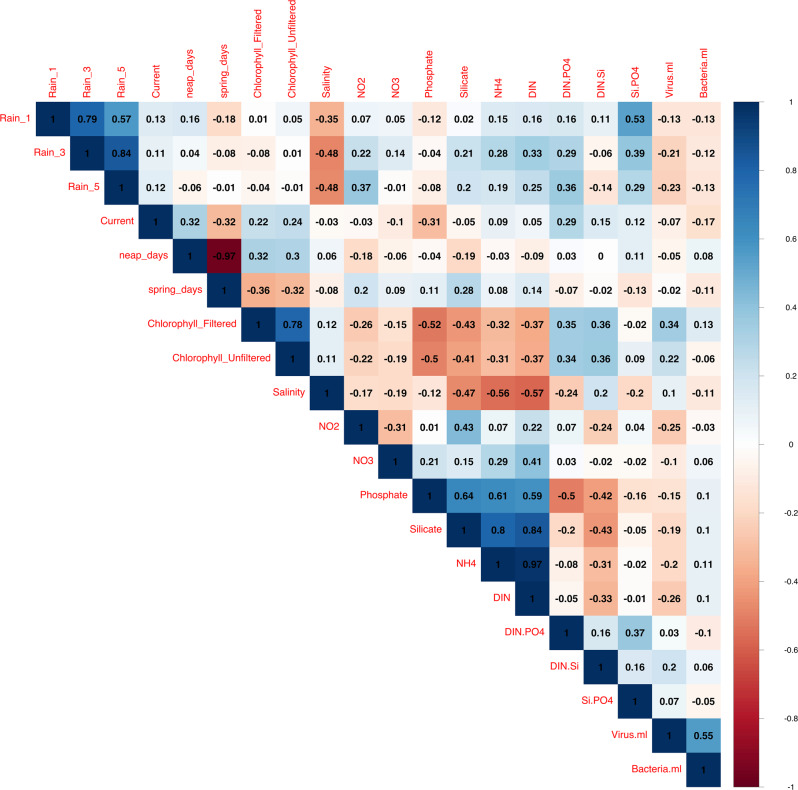
Correlation of various biotic and abiotic factors for all sites. Correlation plots showing Pearson correlation values of all biotic and abiotic factors measured during the study.

Pearson correlation analyses were conducted between environmental parameters, as shown in Fig. 1. A notable correlation interaction can be observed in pairs between the tides, chlorophyll and nitrogen ratios (DIN:P and DIN:Si). Higher chlorophyll concentrations were observed during spring tides when tidal-driven mixing was the highest. Vice versa, a lower chlorophyll concentration was observed during neap tides. Interestingly, chlorophyll concentrations were positively correlated with the N ratios but not with the raw N values. The rain also brought higher N ratios, which was unexpectedly not followed by the increase in chlorophyll concentrations.

**Fig. 2:**
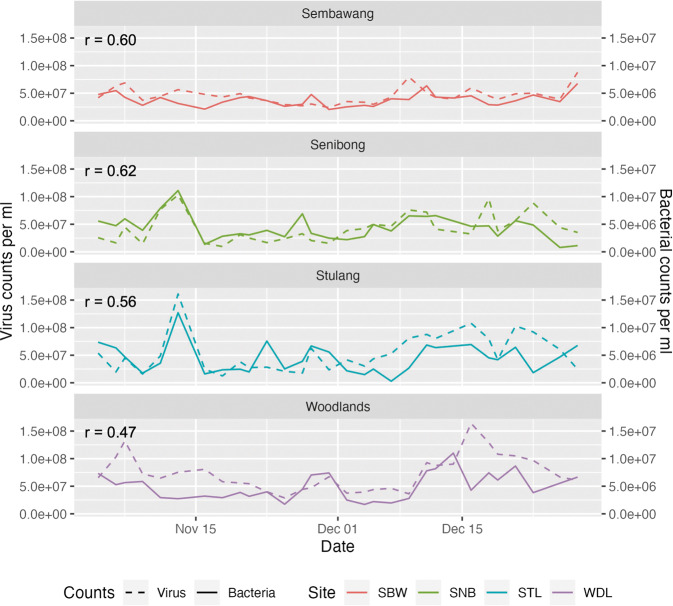
Flow cytometry counts of bacterial & virus-like particles. The line plot shows the high degree of correlation between bacteria and viral abundance in all 4 sampling sites.

Across the domains, viral counts showed a positive correlation with bacteria, in both the FCM gated counts and chlorophyll concentrations: high viral counts were found when high bacterial counts were also observed, and vice versa. This is further seen in Fig. 2 where the viral and bacterial FCM counts from nearly all points were in sync with each other in their increases and decreases.

### Trajectory of community succession

**Fig. 3:**
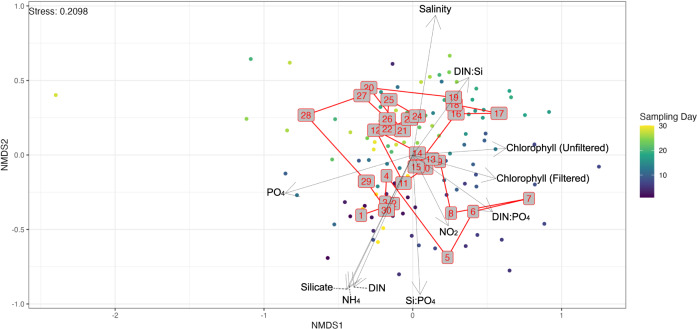
Non-metric multidimensional scaling (NMDS) on the Bray-Curtis distance between each sample. Arrows show *envfit* results, while the red numbers in grey boxes show the centroids of each sampling day. The overlapping *envfit* arrows located around (−0.45, −0.85) are DIN, NH_4_, and Silicate measurements.

Dimensionality reduction of the Bray-Curtis distance using non-metric multidimensional scaling (NMDS, Fig. 3) which shows the community succession between one sampling day and the next seemed to revolve around a common stable state, circling the midpoint of (0,0). Furthermore, *envfit* analysis revealed many water quality parameters that were highly correlated with the samples. No significant autocorrelation of environmental parameters were observed (Fig. S6).

Principal components analysis (PCA) returned the variables that contributed heavily to the variation in the data in attempt to reduce its dimensionality (Fig. S7, Table S4). The first axis represents 40.6% of the variation found in the data, which was mostly contributed by two ASVs related to *Cyanobium* PCC 6307 (88.49%). The second axis represents 13.77% of the variation, which is mostly explained by changes in the abundance of two *Cand*. Nitrosopumilus ASVs (82.08%). These two taxa represented the organisms that explained most of the variation in our time series.

**Fig. 4:**
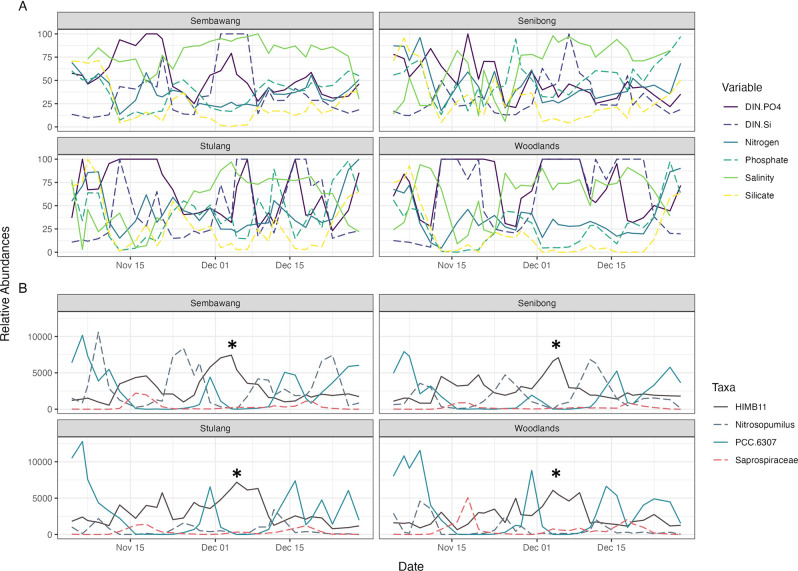
Time-series of nutrients and 4ew of the most abundant taxa, note the changing peaks of the nutrient availability and dominant taxa. **A** Nutrient data have been normalised between 0 to 100 to set everything on the same scale and order of magnitude for easier comparison. **B** Amplicon reads have been normalised to the median total read count. The asterisks (*) note down the position of the HIMB11 peak mentioned in the main text.

Canonical correspondence analysis (CCA) showed how each ASV might have been influenced by nutrients as well as the salinity of the water (Fig. S8), with summarising the sensitivity of a few notable taxa. To illustrate this further, Fig. 4 shows a time series of these taxa and the relative concentrations of nutrients for comparison. No taxa seemed to become dominant in the community for an extended period of time; as the abundance of one species rose, others fell, and vice versa. For example, the small peak of HIMB11 after the 1^st^ of December was characterised by the lower abundance of other taxa, and coincided with the relatively high DIN:Si, Salinity, and DIN:PO4.

Our results were validated against SBW samples from Chènard et al. (ref. 11) to show the dynamic community resilience over longer periods of time. After adding the monthly SBW samples from Chènard et al. (ref. 11), all 4 sites still displayed a distribution comparable to Fig. 3 but with an additional cluster of monthly SBW samples. The probable artifact may be attributed to a batch-effect (Fig. S9) because samples had been collected with a different methodology. Comparison of bar plots between phyla across the two time series, as well as ampvis barplot analysis demonstrate that the median abundance value of each family in the bi-daily time series falls comfortably within the range of the monthly time series, and vice versa, with differences in relative abundances of each family attributed to the differences in environmental variables in the two time series (Fig. S10). Differential abundance analysis was performed with *DESeq2* to identify the extent of the batch effect and identified only 29 genera, belonging to the rare members of the community, that were significantly different between the monthly and the bi-daily samples (Fig. S11). Following batch correction, we noticed that the bi-daily data appears to orbit around the central point (0,0), as illustrated in Fig. 3, while the monthly data from Chènard et al. (ref. 11) had no discernible pattern or direction. (Fig. S13).

### Network analysis

Co-occurrence networks show potential interactions between taxa, such as predatory (−), parasitism (+), symbiosis (+), and antimicrobial (−) relationships. However, networks could not differentiate between the different types of interactions; they do not make a distinction between parasitism and symbiosis as both are positive relationships. Furthermore, it is very difficult to experimentally confirm these relationships, and thus co-occurrence networks are more useful to generate hypotheses rather than to test them [49–51].

Despite its many limitations, one interesting point can be noted from the results of our co-occurrence network in Fig. S14. The family Saprospiraceae (especially ASV0037) has an Eigenvector Centrality of 1, meaning that its node is well connected to other well-connected nodes, making it a very influential node. As shown in Fig. S15, the phylogenetic tree developed from Saprospiraceae 16 S rRNA genes extracted from the NCBI RefSeq database and amplicons in this study, showed that the most abundant Saprospiraceae-related ASV (ASV0037) was found to be most closely related to *Aureispira maritima*, a motile Saprospiraceae first isolated from barnacle debris in Thailand [52].

## Discussion

### Stability of Johor Strait microbial community

While numerous studies have been conducted on disturbances and community resilience, many of them focus on a single disturbance event [2, 53]. Philippot et al. (ref. 2) has suggested to separate compounded disturbances into individual components, including their properties, intensity, frequency, and order of occurrence.

At our study site in the Johor Straits, isolating these disturbances is unfeasible, as they repeatedly unfold in a random, interconnected pattern, in some instances even occurring simultaneously. Yet, during the duration of the study, the community structure remained in a relatively stable state, with no single taxa becoming dominant for an extended period of time during the sampling period, despite the frequent perturbations experienced in the area (i.e., irregular nutrient-enriched freshwater pulses from various sources, the temporally consistent saline seawater intrusion that comes with the tidal currents, among other factors) (Fig. 3).

Since bottom-up disturbances from abiotic factors are all naturally-occurring phenomena that can be safely assumed to have been occurring long before sampling, we propose that repeated disturbances may have driven selection, adaptation, and/or diversification of the community. As a result, the microbial community that we observe today is resilient and able to tolerate these frequent disruptions. A history of repeated disturbances may promote community resilience through these methods, according to earlier studies and evaluations [1, 2, 54, 55].

Moreover, comparison with samples taken from a similar location three years prior further supports that low resistance of the microbial ecosystem (Fig. S13) is inherent and complementary to its resilience. While perturbations do not cause massive, bloom-level changes, they do affect the microbial community structure to a certain extent with multiple similar, but not identical, stable communities. A clear succession path for the bi-daily data in Fig. S13 shows that the bi-daily sampling is able to capture the effect of individual disturbances, i.e. the disturbance happening over a ~2-day time scale. Nevertheless, both the monthly and bi-daily sequencing data show similarly stable communities, i.e. none of the samples are bloom samples, which may reveal that the stable state (where no blooms happen) varies quite widely: the microbial ecosystem is highly resilient towards changes to become a bloom-dominated state, but it has a low resistance towards changes in community composition in a non-bloom state.

The most common taxa in the Johor Strait (i.e. PCC6307, HIMB11, cand. Nitrosopumilus) alternately became numerically dominant, but none of them ever dominated the community for an extended period (Fig. 4). This suggests that the changing environmental conditions constantly reshape the available niches (*sensu* Hutchinson (ref. 56)) and, as a result, rebalance the abundance of each taxon. Non-dominant populations persist in low numerical abundance, until new conditions favour their growth. The changing peaks in Fig. 4 are mostly made up of organisms related to HIMB11, *Nitrosopumilus*, PCC6307, and on a few instances, Saprospiraceae. Each population would need unattainable rates of growth, acclimation, and adaptation to keep up with the ever-shifting environmental conditions and nutrient availability. Essentially, Fig. 4 showcases the rapid emergence of slightly different niches that are promptly filled by the most suited and quickest colonizing population.

### Top-down controls

#### Predatory/algicidal bacteria

By having an Eigenvector Centrality of 1, Saprospiraceae is a potential ‘keystone taxa’ in the microbiome of the Johor Strait, that is, it has the potential to have a crucial role in community structuring. Keystone taxa do not need to be the most abundant species in the community; there are documented instances where rare microbial taxa in the microbiome are disproportionately important for the well-functioning of the entire ecosystem [57].

Based on our phylogenetic tree (Fig. S15), the most abundant ASV related to Saprospiraceae (ASV0037) was found to be most closely related to *Aureispira maritima (NR_041537)*. *Aureispira*-related strains have been found to exhibit algicidal and predatory activity [58], making ASV0037 a potential top-down controller of the Johor Strait microbial community. In general, many members of the family Saprospiraceae were found to have algicidal or predatory properties [58]. Thus, some studies have suggested to use *Saprospira*-like organisms to control cyanobacteria blooms, but this has never been experimentally shown nor attempted [58–60].

#### Viruses

One other source of top-down control are viruses. What viruses lack in biomass, they make up for in abundance [61]. As viruses lack their own machinery for reproduction, they hijack bacterial cells and reproductive abilities for their replication [62]. This intrusion releases a confetti of new virions, various cellular parts, and dissolved organic matter in the process. In this way, viruses relate the top-down controls of a community by predation to the bottom-up controls by way of nutrient availability: marine phages recycle an estimated 20% of all microorganisms every day, therefore making them a major driver in ocean biogeochemistry, and thus its biology and community structure [62].

The positive correlation between viral counts and bacterial abundances (Fig. 2), as well as the fact that no algal blooms nor any microbial abundance that is out of proportions were observed, supports the well-known Kill-The-Winner hypothesis [63]. In this model of phage top-down control of microbial communities, more viruses are infecting the fastest-growing strain of microbes, killing the potential winner. No one species is allowed to dominate the community, which allows other microbes to persist and thus increasing the diversity and resilience of the system. This method of top-down control may be the primary mechanism of keeping the community structure in check.

### Bottom-up controls

#### Limiting nutrient availability

Chlorophyll measurements are a proxy for microbial biomass, especially photosynthetic microbes, and nitrogen limitation has been observed in previous studies in the region [17, 19]. We saw strong positive correlations between chlorophyll and N ratios (N:P and N:Si) that were consistent across all sites (Fig. 1). As the increase in N compared to P or Si also results in an increase in microbial biomass, we also suggest that nitrogen is the limiting nutrient in preventing the over-proliferation of photosynthetic microorganisms.

### Interplay between biotic and abiotic factors controlling algal blooms in Johor Strait

HAB species that were detected in previous studies [8, 16, 19–21, 48] were also observed in 81 out of 118 samples in our time series. Similar to the more common taxa alternating more rapidly in the Johor Strait (i.e. PCC6307, HIMB11, *cand. Nitrosopumilus*), these HAB taxa occur in very low numbers. However, the latter were not observed to dominate during the course of our sampling. Thus, we propose that a HAB event may develop only when two specific conditions are met: [1] an open niche is available and [2] top-down controls are no longer controlling the HAB population.

Two lines of evidence support the above hypothesis. Firstly, our results show that despite rapid and significant changes in the environmental conditions, the overall community structure recovers rapidly towards a stable centre-point. Moreover, changes in environmental parameters alone do not cause the microbial ecosystem to crash and turn into a HAB-dominated community, showing the community’s resilience towards disturbances. Secondly, there is strong evidence for the community to be controlled from the higher trophic levels by predation and viral lytic activities. While we do not have data on larger predators such as copepods and other zooplankton, viruses show changes that are synchronized with the variations in the bacterial populations (Fig. 2).

The dominant species in the Johor Strait microbial community was shown to alternate between a few species depending on the nutrient conditions of the water (Fig. 4). Changing environmental factors, coupled with the fact that HAB species were nearly always present in low amounts in the water, support the hypothesis that a HAB event may happen due to an available, open niche. A 2019 Singaporean study had proposed something similar: an observed dinoflagellate *Karenia* bloom may have happened due to a niche being opened whereby the abundance of diatoms had previously decreased [20].

While the system seems to be able to adapt to changes in nutrient availability (bottom-up controls), it does not show adaptations to changes from the top-down (predators). We speculate that this may be because top-down controls are more reactive in nature than causative; top-down controls do not initiate changes in the organisms at lower trophic levels, rather, top-down controls respond to the varying abundance of organisms at the lower trophic levels. When bottom-up controls make way for an open niche, HABs may be prevented from happening by the presence of predators limiting the uncontrolled growth of the HAB species. However, if their growth is somehow not inhibited by the top-down controls, the HAB species may take over and eventually dominate the microbial community, and thus leading to an occurrence of a HAB. This top-down control of algal blooms has been reported in several studies, be it the onset of blooms due to the decrease in grazer populations [64–67] or the termination of blooms due to viral lytic activities [62, 68–71]. Irigoien et al. (ref. 67) have also proposed the “loophole theory” in which blooms are formed when they are able to find a ‘loophole’ and escape the grazing control. Finally, the Oksanen–Fretwell theory [72] suggests that highly productive environments, such as the Johor Strait, should be under top-down control, therefore the growth of bloom organisms should be limited by predatory bacteria and viruses rather than nutrient availability.

In the Johor Strait, the performance of these top-down controls may be affected by two mechanisms, that is, the low rates of water mixing in the Johor Straits and the high ammonia concentration in the water. As mentioned in the section above (Results – General overview), water mixing in the Johor Strait is primarily driven by tides (Fig. S4). Previous studies have also concluded that blooms happen more frequently during neap tides when tidal mixing is at its minimum, due to the higher cell densities during a stable water column [17, 19]. However, we propose that stratification during minimum tidal mixing may also prevent the predators (grazers, viruses) from reaching their target preys. Sufficient tidal mixing would prevent the isolation of HAB species, thus exposing them to predators and inhibiting the HAB species’ proliferation when they find an open niche.

Secondly, ammonia (NH_4_) is traditionally known to be toxic for aquatic grazers such as copepods and other zooplanktons [73–77]. Grazers perish from the toxicity when NH_4_ concentrations rise above a certain concentration, whose 48-hour LC50 has been observed to be anywhere between 0.17 mg/L and 2.94 mg/L depending on the species and maturity of the copepods [73, 75–77]. NH_4_ may enter the water through runoff from land, sewage, and industrial water discharge. Knowing that the Johor Strait is bounded by two highly urbanised regions with anthropogenic nutrient inputs [6–10], this source of grazer mortality is not entirely improbable.

### Limitations & further study

Sampling was conducted every other day to stretch resources to cover a sampling period of 2 months. Therefore, any change with a resolution of less than 2 days could not be observed, thus limiting our study. Furthermore, we have been focusing on compositional rather than functional resilience, which could have distinguished between active and inactive bacteria. Future research in this direction will reveal which genes are activated under various conditions in response to specific disturbances and determine whether these genes are, in fact, contributing to the dynamic resilience of Johor Strait and the key to mediating the effects of the perturbations.

## Conclusion

Frequent disturbances may have shaped the Johor Strait microbial community by selection, adaptation, and diversification of organisms that can withstand these perturbations. The result is a diverse and relatively-stable community, adapted to living in a constantly changing environment. The increased importance of top-down controls in a highly productive environment such as the Johor Strait is in accordance with the Oksanen–Fretwell (ref. 72) theory, where the growth of bloom organisms is limited by predatory bacteria and viruses. Blooms may happen when an extreme disturbance is introduced into the system and changes both bottom-up or top-down controls, i.e., when an available empty niche temporally coincides with the demise of grazers or viral predators that keep the bloom populations in check. While we were not able to completely disentangle the effects of the different disturbances, this study further highlights that complex interactions between perturbations play a key role in regulating microbial community structure.

## Supplementary information

Supplementary information.

## Data Availability

Raw sequencing data have been deposited to SRA under Bioproject number PRJNA848014 for the bi-daily data and PRNA929445 for the monthly data. Processed data and scripts are available on https://github.com/winanonanona/2020-Time-Series.
