# Supplementary material for: Frequent pulse disturbances shape resistance and resilience in tropical marine microbial communities
Source: ISME Commun. 2023 Jun 6;3:55. doi: 10.1038/s43705-023-00260-6 (PMC10244338; doi:10.1038/s43705-023-00260-6)
Supplement: Supplementary file 1 — Supplementary information. [file 43705_2023_260_MOESM1_ESM.pdf]

Table S1 Details of sampling sites

Site	Coordinates	E/W	N/S	Time of sampling
Sembawang (SBW)	1.4643, 103.8372	East	South	9.30am
Senibong (SNB)	1.4846, 103.8170	East	North	9.30am
Woodlands (WDL)	1.4554, 103.7790	West	South	11.00am
Stulang (STL)	1.4665, 103.7791	West	North	11.00am

Table S2 Sembawang Tide station measures tides at 1.830m below the Singapore Height Datum, which is 1.652m above the Chart Datum. Along the course of our sampling, the highest tide height measured at 3.74m, the lowest at 0.04m, and the average tide height was 2. This table is adapted from the Singapore Tide Tables Year 2020, published by the Marine and Port Authority of Singapore in 2019.

Sembawang Tide Station	Height (m)
Mean High Water Springs	3.1
Mean High Water Neaps	2.5
Mean Level	1.9
Mean Low Water Neaps	1.4
Mean Low Water Springs	0.7

Table S3 Dates of spring and neap tides, and their related moon phases

Moon Phase	Tide	Date (dd/mm/yyyy)
Full moon	Spring tide	31/10/2020
Last quarter	Neap tide	08/11/2020
New moon	Spring tide	15/11/2020
First quarter	Neap tide	22/11/2020
Full moon	Spring tide	30/11/2020
Last quarter	Neap tide	08/12/2020
New moon	Spring tide	15/12/2020
First quarter	Neap tide	22/12/2020
Full moon	Spring tide	30/12/2020

Table S4 Summary of PCA axis contribution and feature importance, (**Figure S7**)

Axis 1 Contributions (40.6%)		Axis 2 Contributions (13.77%)	
ASV0004 (PCC-6307)	69.69%	ASV0006 *(Cand. Nitrosopumilus)*	51.87%
ASV0008 (PCC-6307)	18.80%	ASV0015 *(Cand. Nitrosopumilus)*	30.21%
ASV0019 *(Cand. Actinomarina)*	1.11%	ASV0003 (HIMB11)	9.67%
ASV0003 (HIMB11)	1.05%	ASV0004 (PCC-6307)	1.71%
Others	9.35%	*Others*	6.54%

Table S5 Summary of CCA results (**Figure S8**) of the top few taxa, i.e. the location on each arrow where a line perpendicular to the arrow and the ASV can be drawn.

Taxa	ASV No.	DIN	Si	PO4	DIN:PO4	DIN:Si	Salinity
(Genus) HIMB11	3	Mid-low	Mid-low	Mid-low	Mid	Mid-high	Mid
(Genus) PCC-6307	4, 7	Mid-high	Mid-high	High	Mid	Low	Mid-low
(Genus) Nitrosopumilus	9, 16	Low	Mid	Mid-high	Low	Mid	Mid-high
(Family) Saprospiraceae	37	Mid-high	Low	Low	Very high	Very high	Very low

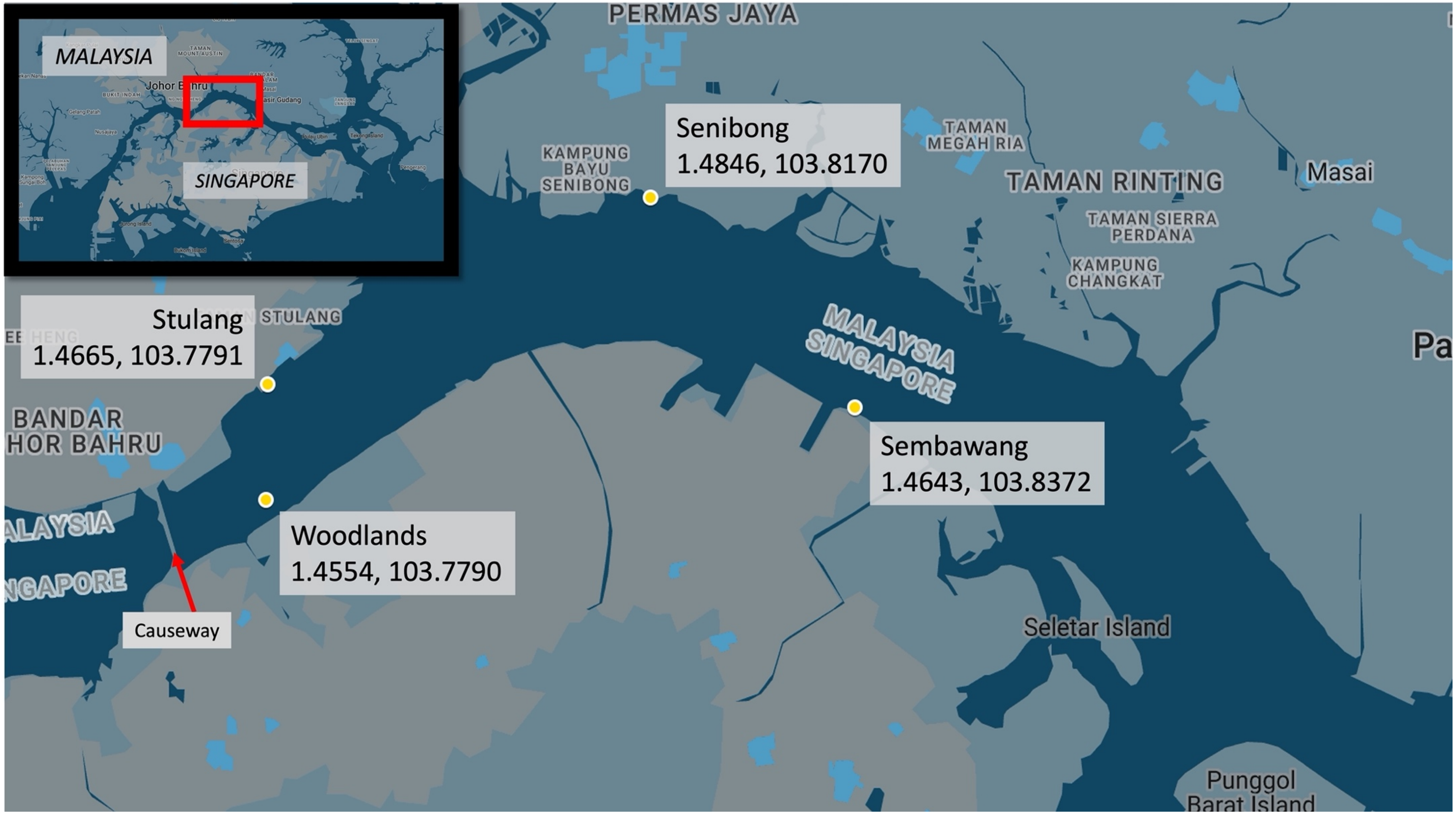

Figure S1 Map of Singapore showing the four stations sampled in this study (coordinates reported in **Table S1**).

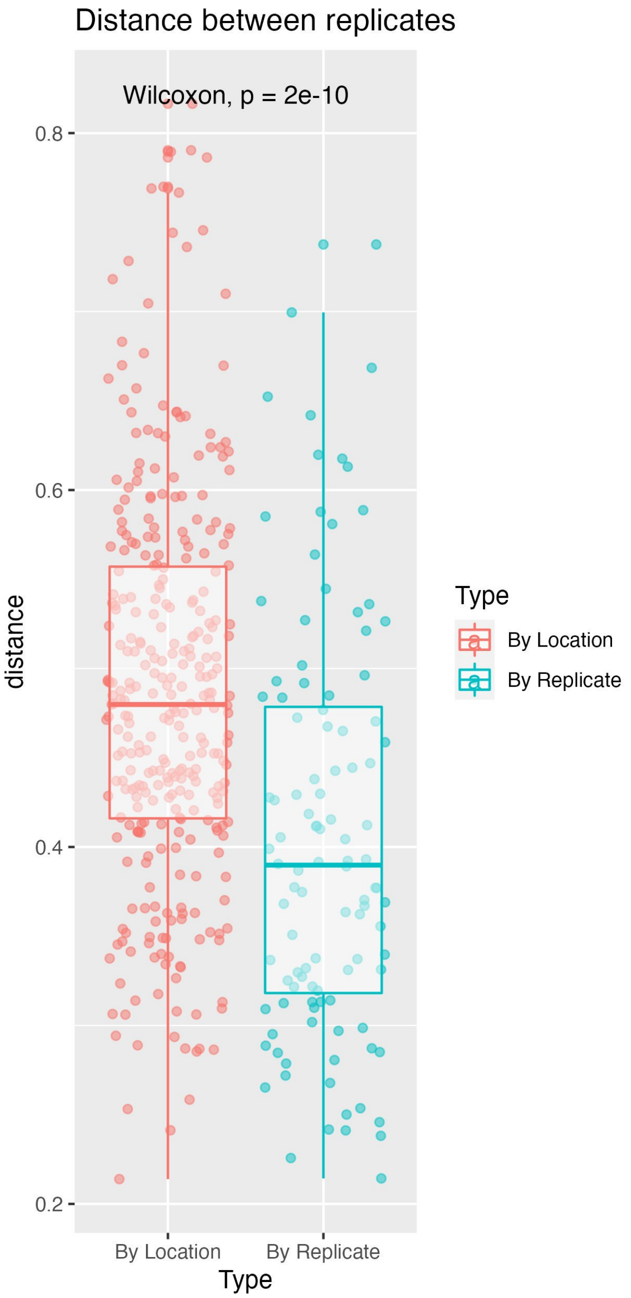

Figure S2 Bray-Curtis distance between different locations on the same day and different technical replicates. The distance between technical replicates is smaller than between locations.

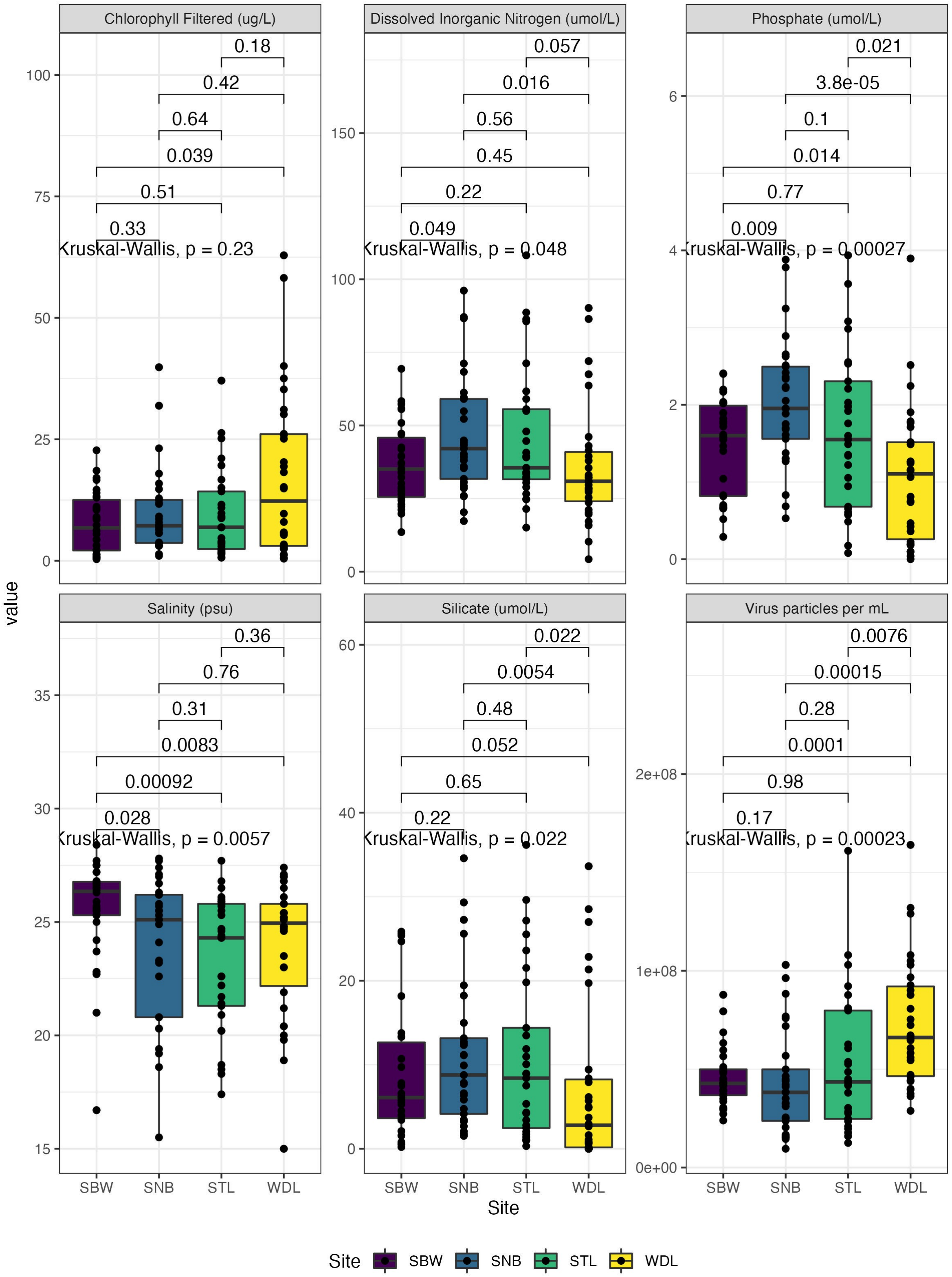

Figure S3 Summary of dissolved nutrient values and environmental parameters. Using Bonferroni correction for multiple comparisons, only p-values below 0.00833 show significance.

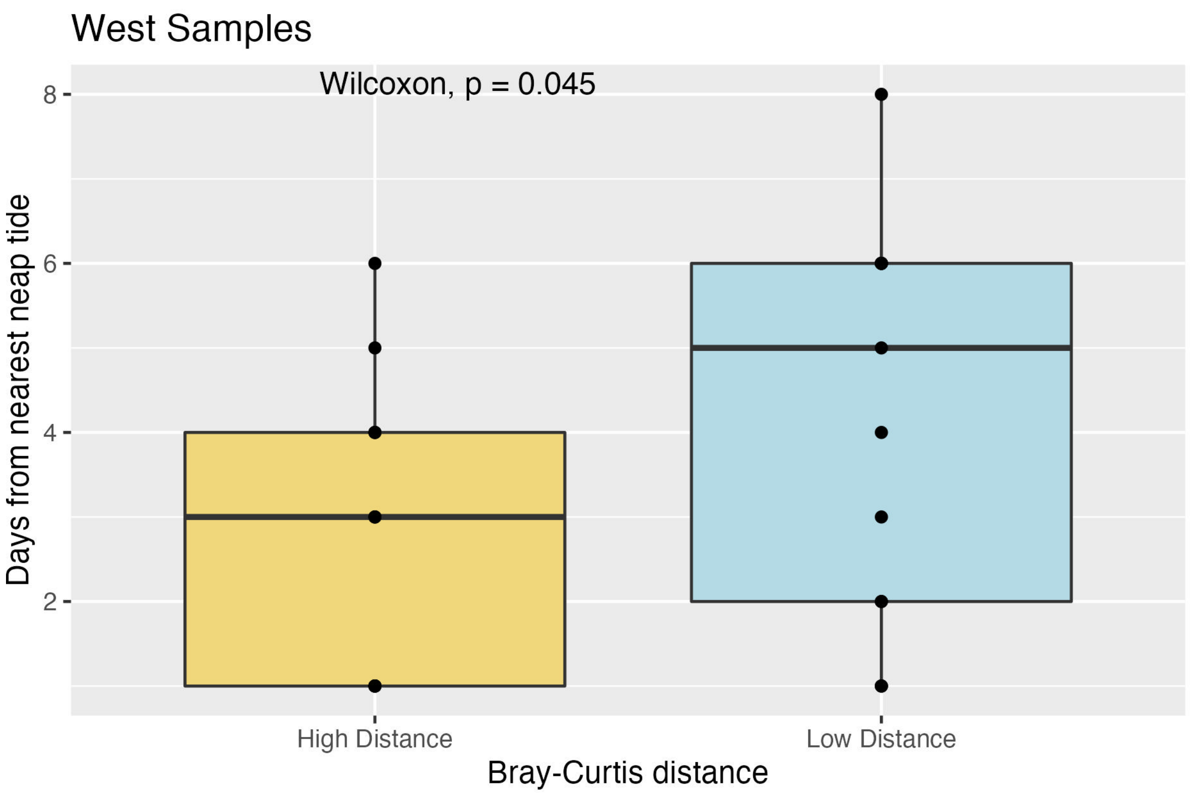

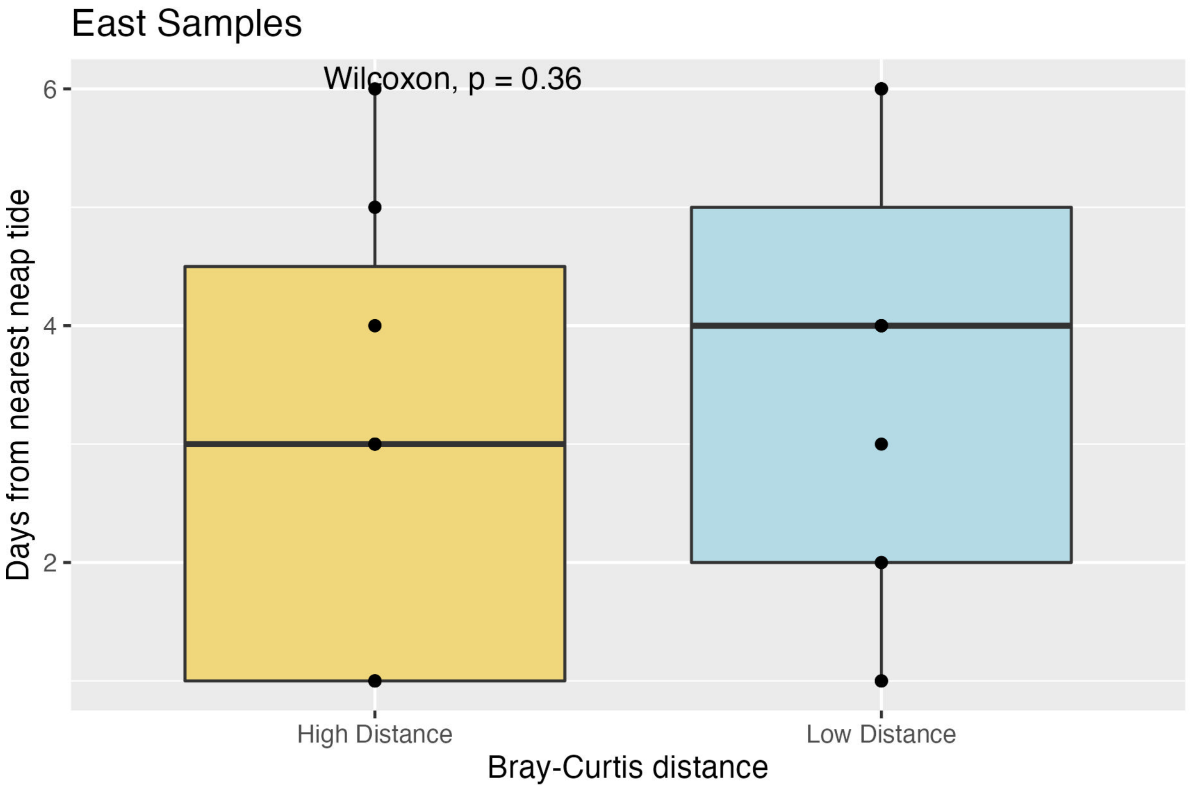

Figure S4 Number of days from the peak of the nearest neap tide of 15 samples with the highest Bray-Curtis distance, versus 15 samples with the lowest Bray-Curtis distance, calculated from the daily average. During neap tides, there is less tidal excursion, less tidal current, and thus a lower tidal mixing. Further in time from the neap tide peak, the water experiences higher tidal mixing. There is a significant difference between the high and low distance samples with regard to tidal mixing in the western samples (top), while the difference is insignificant in the eastern samples (bottom). This shows a higher influence of tidal-driven mixing the eastern samples closer to the South China Sea, while tidal mixing in the western samples closer to the causeway only happens during spring tides.

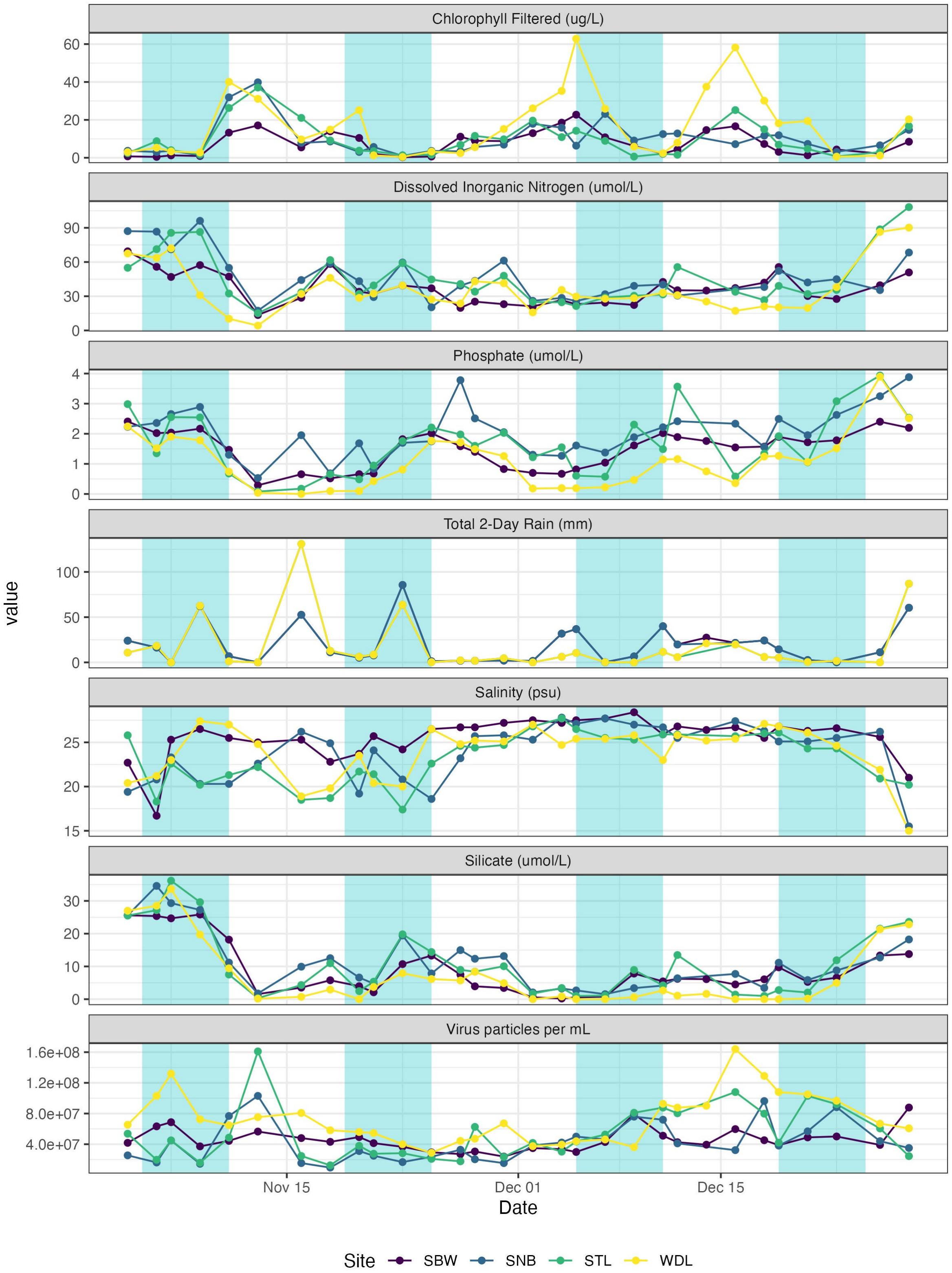

Figure S5 Time-series of biotic and abiotic factors for the four sampling sites. The blue shades highlight 3 days before/after the peak of the neap tide.

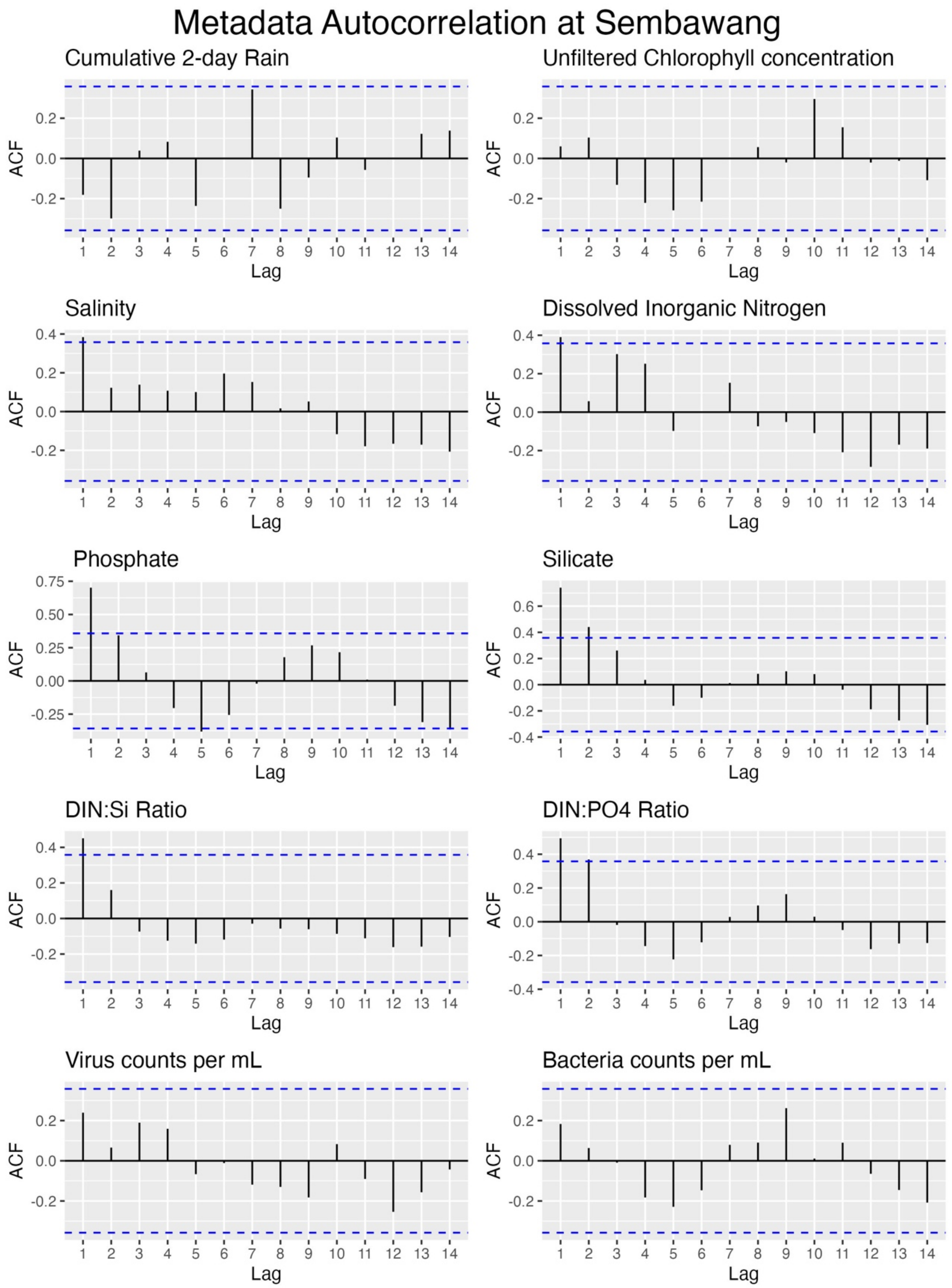

Figure S6 Autocorrelation of the metadata. Blue dotted line indicates the threshold for a significant autocorrelation value. No significant autocorrelation was detected. Only autocorrelations for the Sembawang metadata is shown; autocorrelation plots of Senibong, Stulang, and Woodlands show similar results.

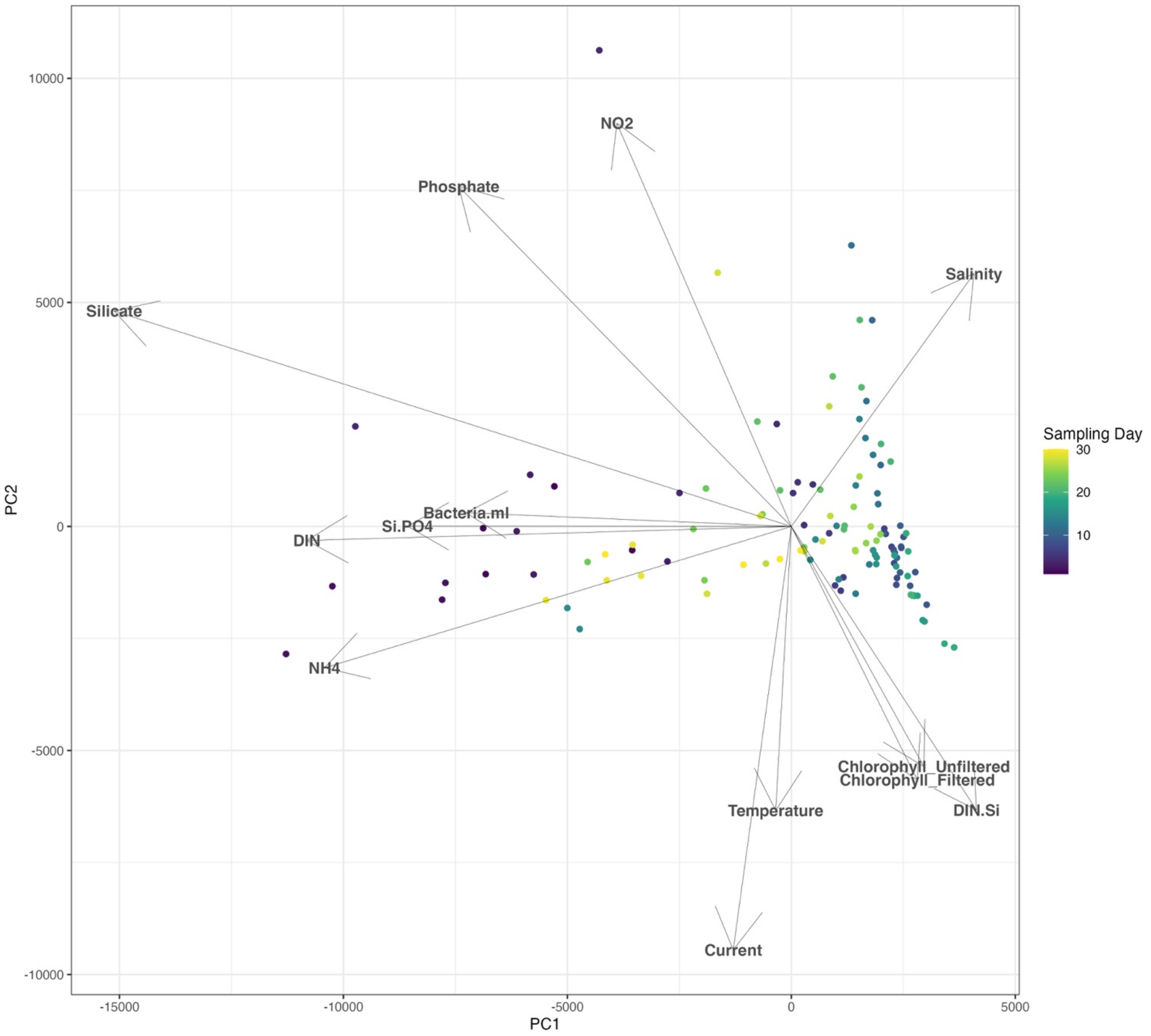

Figure S7 Principal Components Analysis of all the samples, overlayed with environmental data fitted using *envfit*. PC1 and PC2, respectively, explain 40.6% and 13.8% of the variance in the data. A summary of axis contributions and feature importance is provided in **Table S4**.

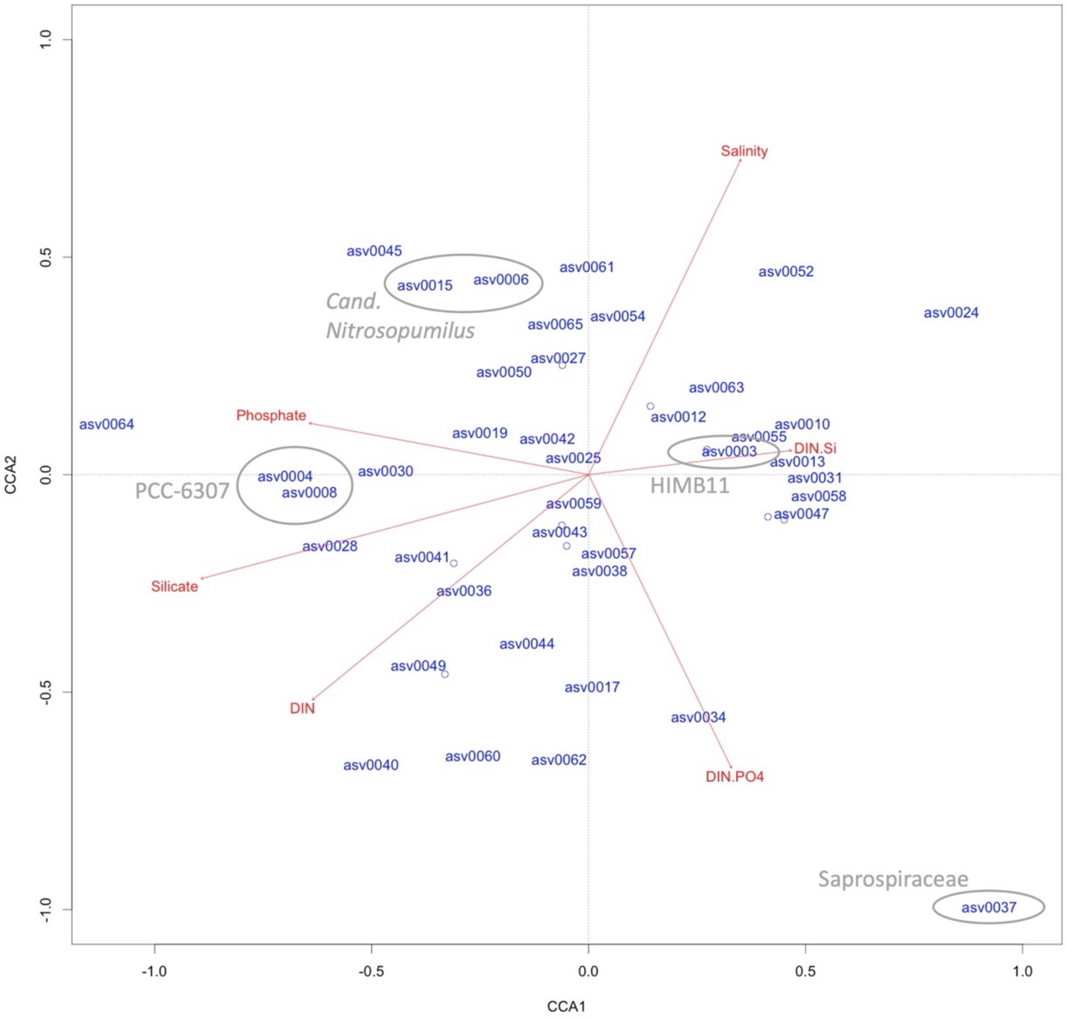

Figure S8 Canonical Correspondence Analysis (CCA) of the top 50 ASVs. The arrows show various environmental parameters. A few notable ASVs has been annotated with their taxonomic classification. summarises each taxon’s relationship to the environmental parameters, i.e. the location on each arrow where a line perpendicular to the arrow and the ASV can be drawn.

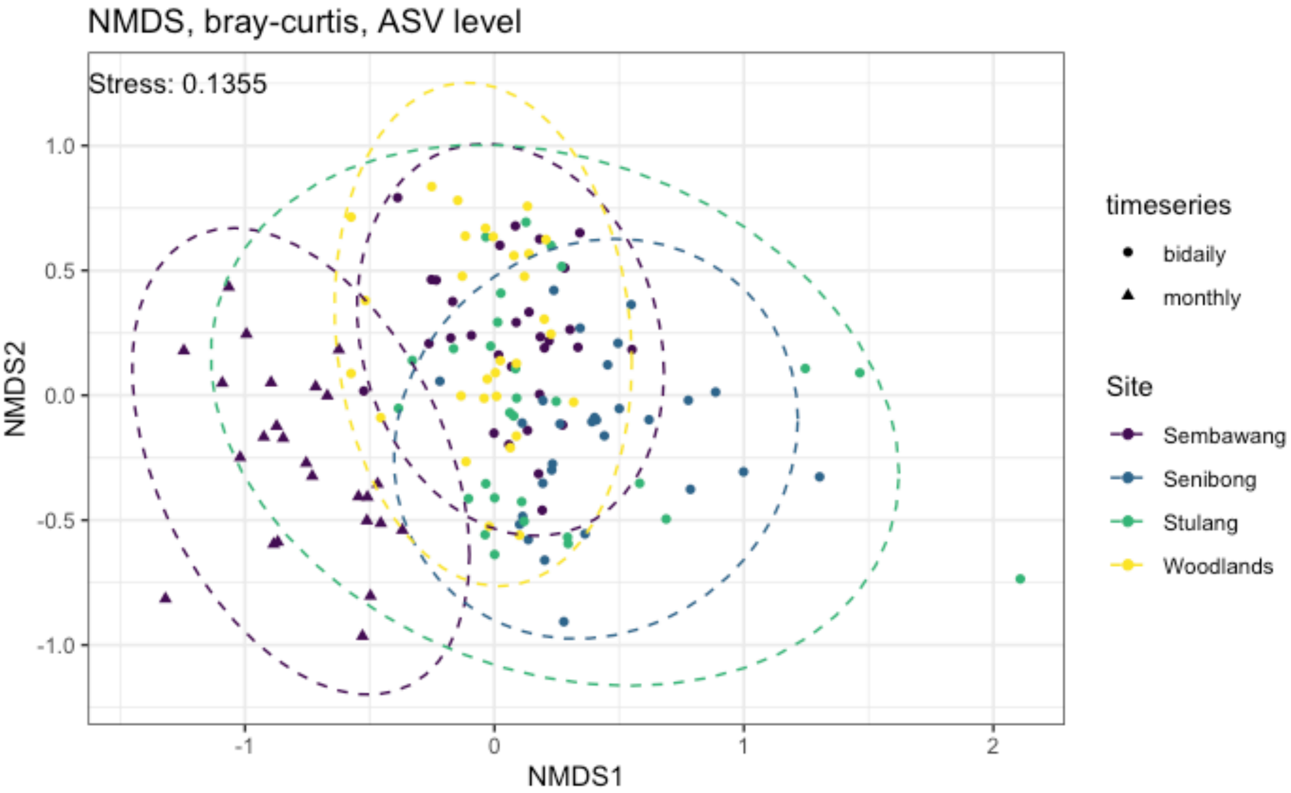

Figure S9 NMDS of the bi-daily sequences from this study and monthly SBW sequences from Chènard (2019)[11]. The bi-daily data show a similar distribution to **Figure 3**, however, the monthly SBW (dark purple triangles) looks as if it was another location altogether. This may be due to batch effects.

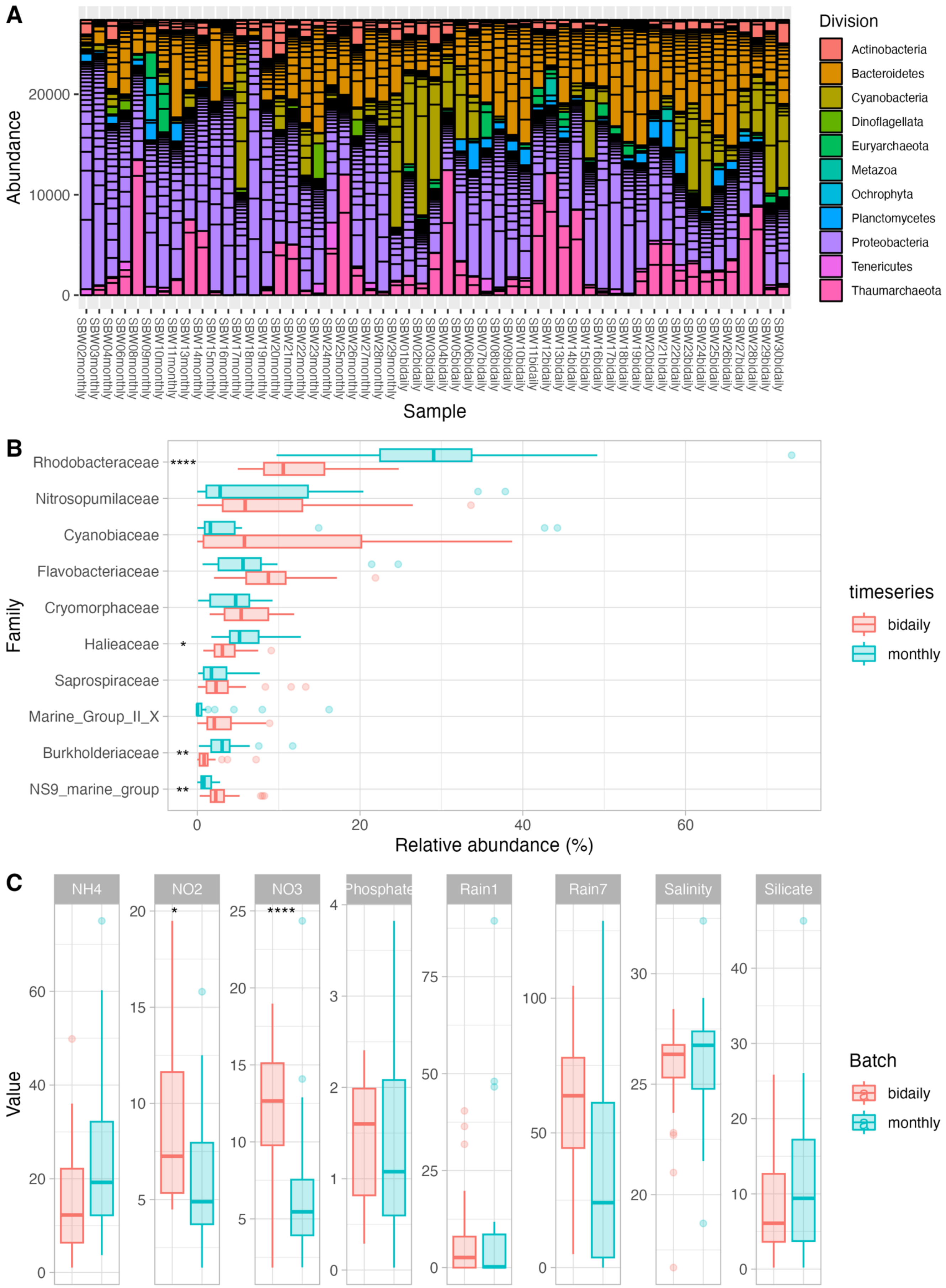

Figure S10 (A) Bar plots of monthly and bi-daily sequences classified at the Phylum level in the SBW samples. **(B)** Boxplot of relative abundances of the top 10 families in the bi-daily and monthly samples. There is generally no difference in relative abundances in the bi-daily and monthly samples, except for

Rhodobacteraceae (p.adj < 0.0001), Halieaceae (p.adj < 0.05), Burkholderiaceae (p.adj < 0.01), and the NS9 marine group (p.adj < 0.01), calculated using a t-test and Bonferroni-adjustment to the p-value. This may be explained by the differences in environmental variables in panel **(C)**. There is significantly higher concentrations of nitrate and nitrate measurements in the bidaily samples than monthly samples (p.adj < 0.05 and 0.0001 respectively), which would account for the differences in the relative abundances of Rhodobacteraceae, Halieaceae, and the NS9 marine group. Rhodobacteraceae (mostly HIMB11) and Halieaceae (mostly the OM60/NOR5 clade) does not utilize nitrate in their metabolism, and thus would be more abundant where there is less nitrate, i.e. the monthly samples. Relatives of the NS9 marine group in Flavobacteraceae use nitrate either for denitrification or nitrate reduction, and thus it is likely that NS9 marine group would be more abundant where there is more nitrate, i.e. in the bi-daily samples. Burkholderiaceae, on the other hand, thrives in low salinity environments. Although the bidaily rain measurements seems higher than the monthly (though not significantly different), the monthly rain data show higher extremes than the bidaily ones. Extreme rain events result in high land runoff entering the Strait waters, and thus higher Burkholderiaceae abundances in the monthly samples.

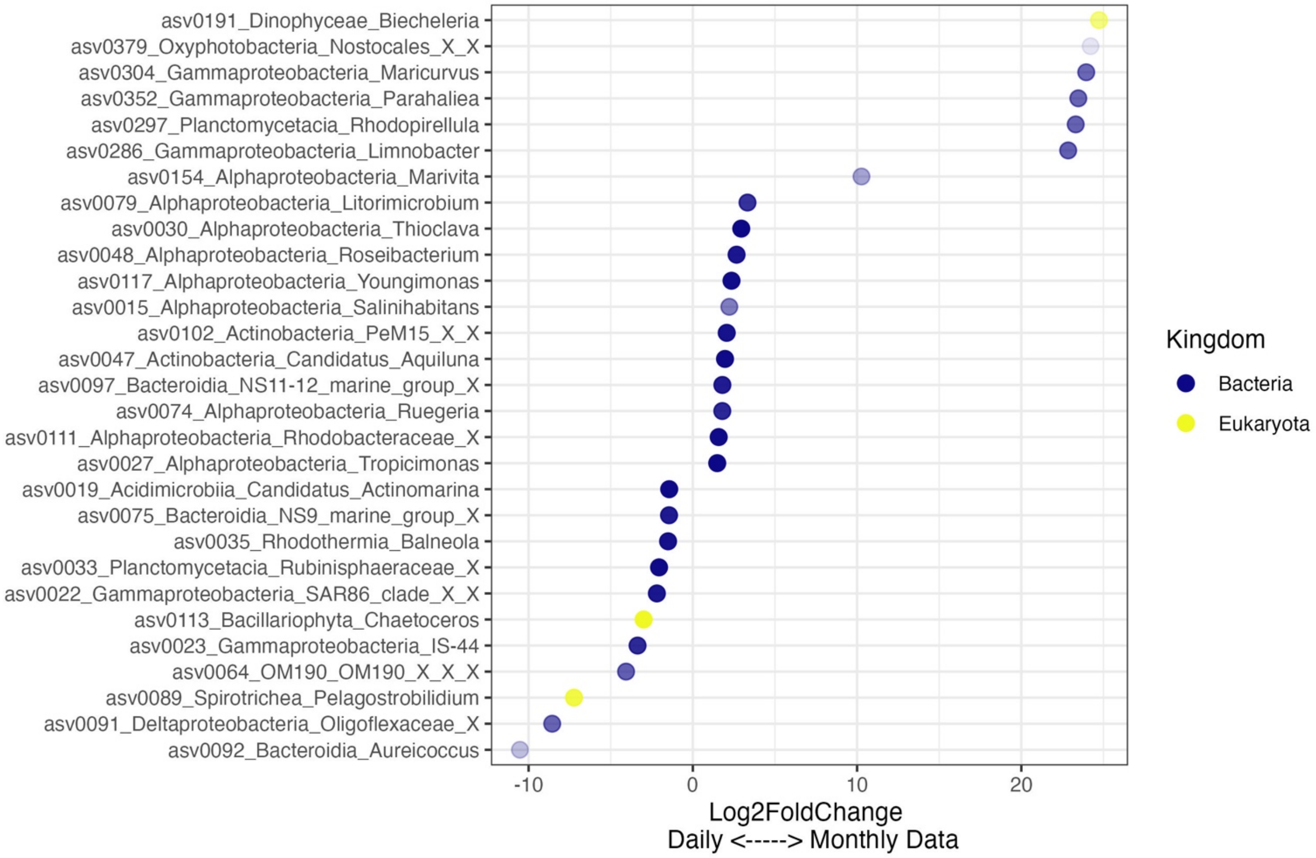

Figure S11 Differential abundance analysis using DESeq2 showing taxonomic differences in monthly & bi-daily data. Many of these organisms are typically associated with harmful algal blooms. Organism names with X-s at the end indicate that these amplicon sequences could not be classified to the genus level.

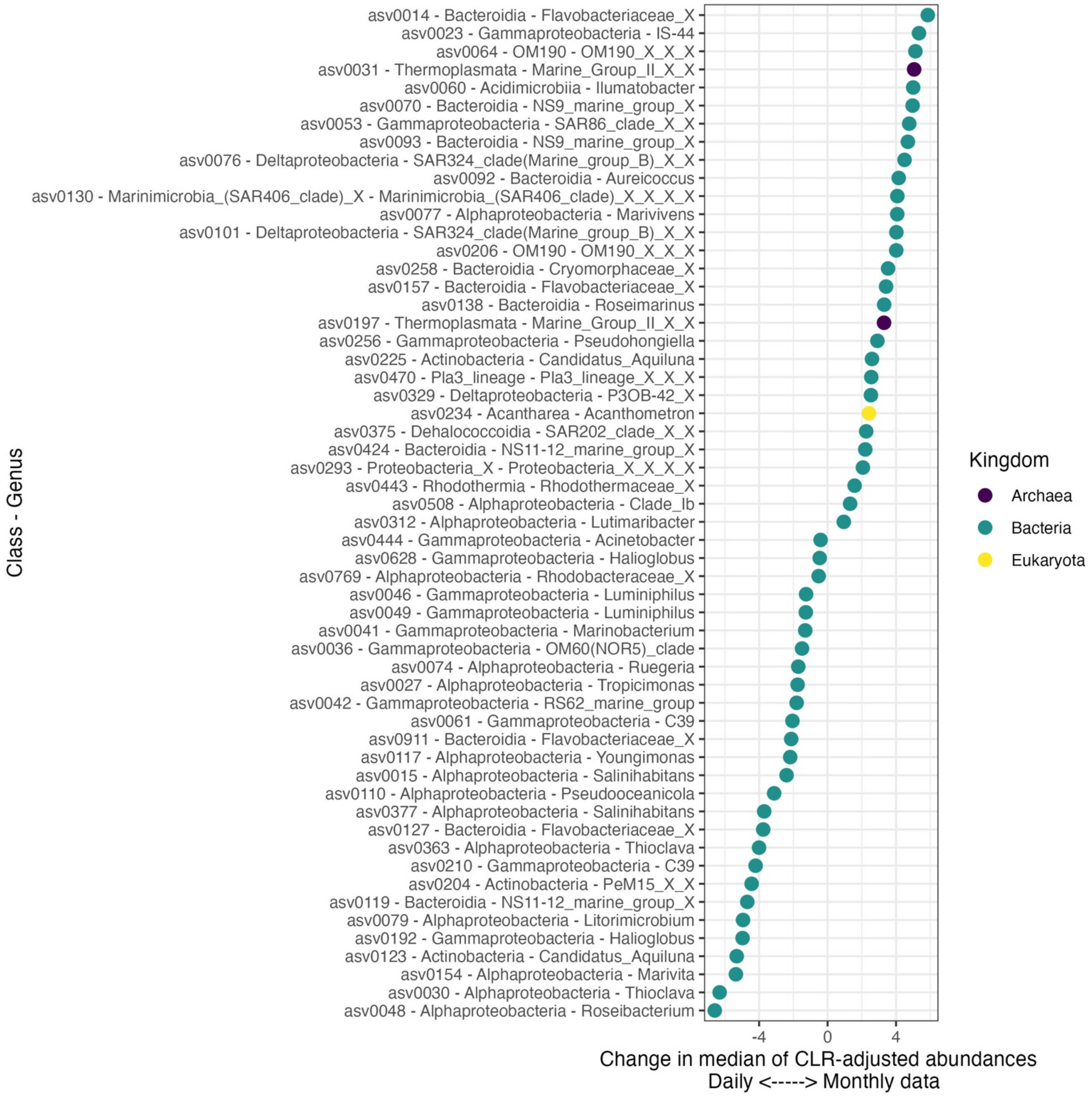

Figure S12 Differential abundance analysis (Wilcoxon-signed rank test on centered-log-ratio-adjusted abundance table, before batch correction) showing taxonomic differences in monthly & bi-daily data. Many of these organisms are also found differentially and significantly abundant using DESeq2. After batch correction, no ASVs are found to be differentially abundant, significantly.

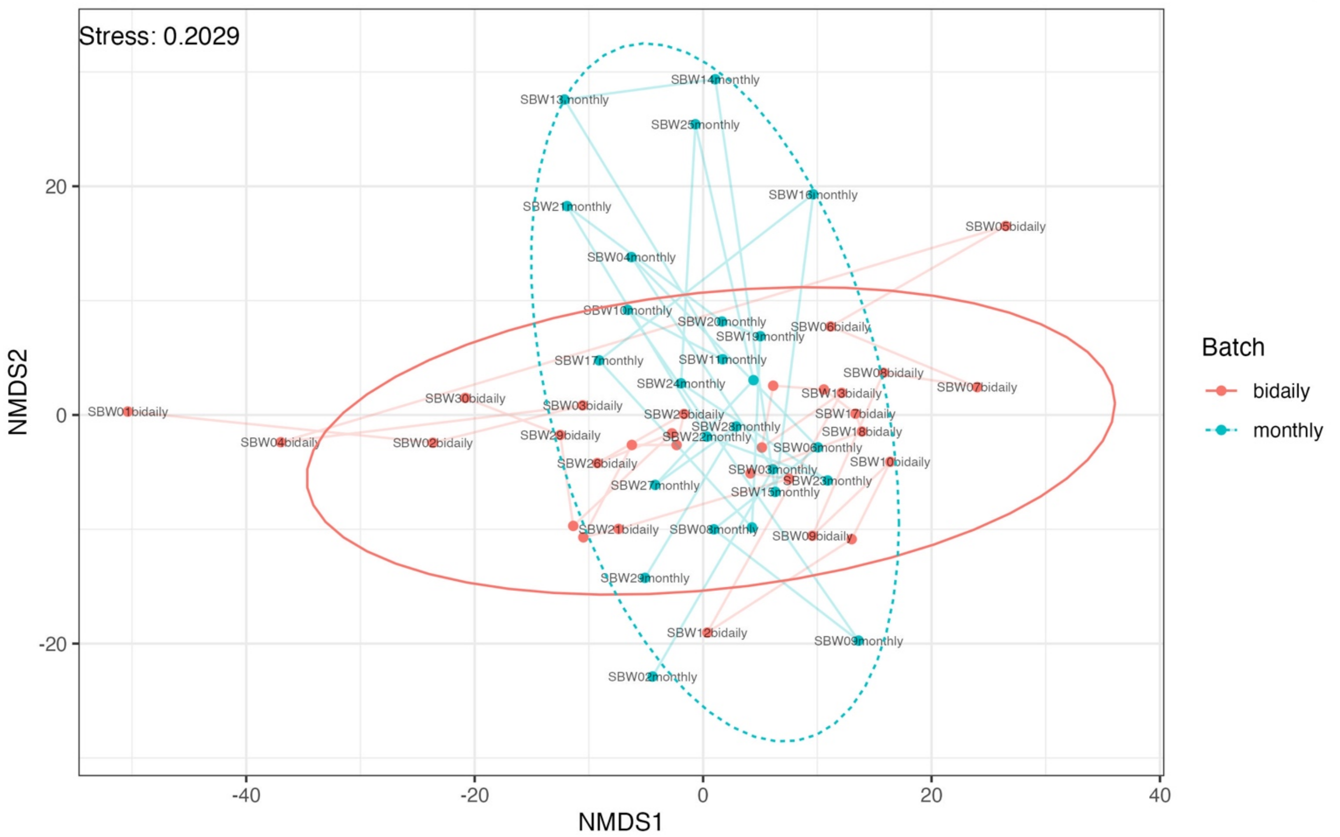

Figure S13 Non-metric dimensional scaling of CLR-transformed, batch-effect corrected data using Euclidean distances. Paths for bi-daily data revolve around the midpoint of (0,0), while monthly data lack any particular direction.

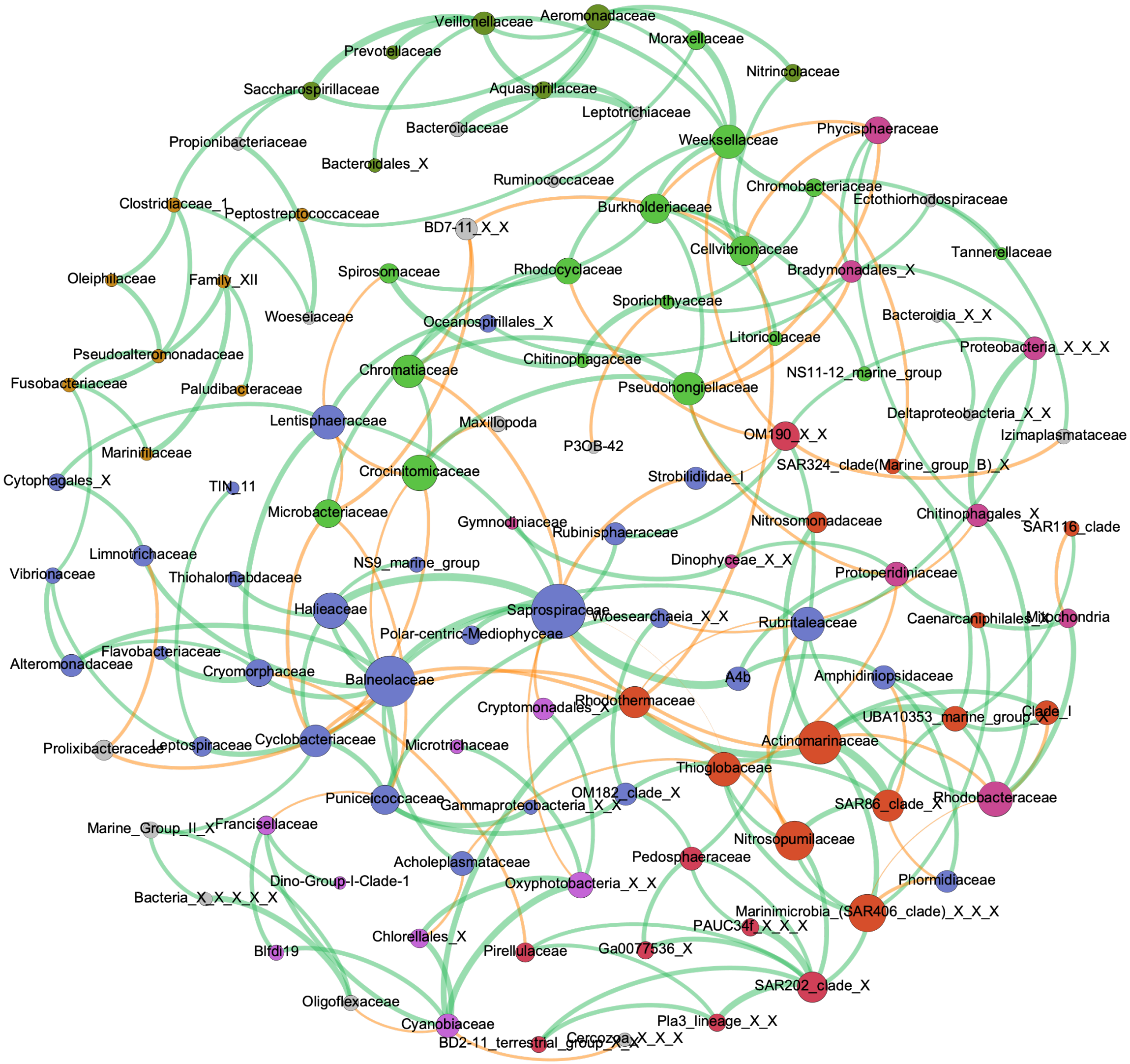

Figure S14 Co-occurrence network analysis at the Family level, showing Saprospiraceae as a potential keystone taxa. Green edges show positive co-occurrence, while orange edges show negative co-occurrence. Colours of the nodes show the potential clusters that co-occur together.

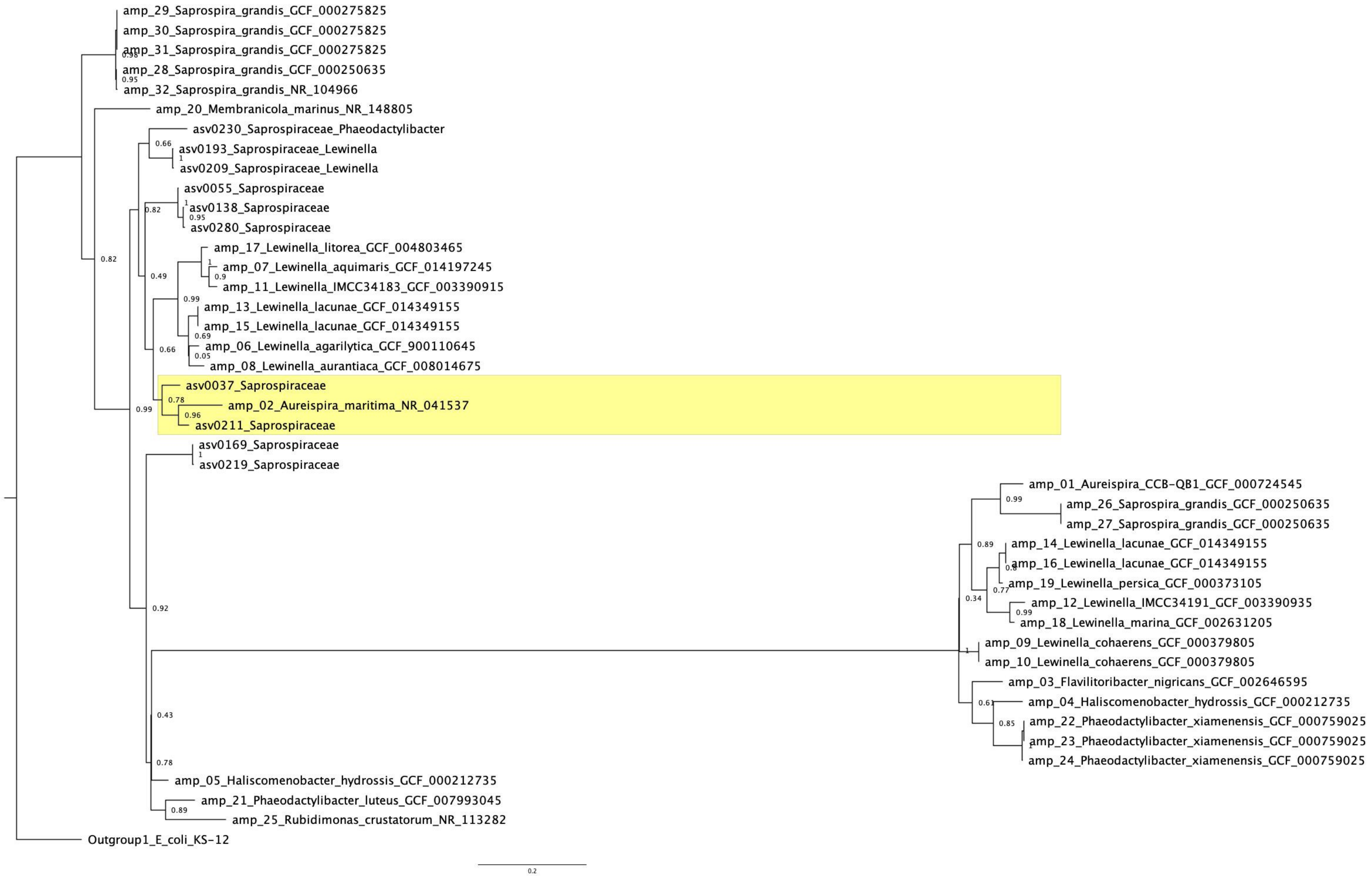

Figure S15 Maximum-likelihood phylogenetic tree showing Saprospiraceae-related ASVs and in silico amplicons taken from the NCBI RefSeq database. Highlighted features contain ASV0037, the most abundant Saprospiraceae ASV, as well as its potentially closest relative, *Aureispira maritima*. Numbers on each internal node indicate the local support values calculated using the Shimodaira-Hasegawa test with 1000 resamples[44]. Codes starting with “GCF” or “NR” are NCBI reference numbers for the genome from each ASV was extracted.
